# Behavioural and neuroanatomical correlates of auditory speech analysis in primary progressive aphasias

**DOI:** 10.1186/s13195-017-0278-2

**Published:** 2017-07-27

**Authors:** Chris J. D. Hardy, Jennifer L. Agustus, Charles R. Marshall, Camilla N. Clark, Lucy L. Russell, Rebecca L. Bond, Emilie V. Brotherhood, David L. Thomas, Sebastian J. Crutch, Jonathan D. Rohrer, Jason D. Warren

**Affiliations:** 1 Dementia Research Centre, Department of Neurodegenerative Disease, UCL Institute of Neurology, University College London, London, UK; 2 Neuroradiological Academic Unit, Department of Brain Repair and Rehabilitation, UCL Institute of Neurology, University College London, London, UK

**Keywords:** Speech, Auditory, Voxel-based morphometry, Primary progressive aphasia, Semantic dementia, Progressive non-fluent aphasia

## Abstract

**Background:**

Non-verbal auditory impairment is increasingly recognised in the primary progressive aphasias (PPAs) but its relationship to speech processing and brain substrates has not been defined. Here we addressed these issues in patients representing the non-fluent variant (nfvPPA) and semantic variant (svPPA) syndromes of PPA.

**Methods:**

We studied 19 patients with PPA in relation to 19 healthy older individuals. We manipulated three key auditory parameters—temporal regularity, phonemic spectral structure and prosodic predictability (an index of fundamental information content, or entropy)—in sequences of spoken syllables. The ability of participants to process these parameters was assessed using two-alternative, forced-choice tasks and neuroanatomical associations of task performance were assessed using voxel-based morphometry of patients’ brain magnetic resonance images.

**Results:**

Relative to healthy controls, both the nfvPPA and svPPA groups had impaired processing of phonemic spectral structure and signal predictability while the nfvPPA group additionally had impaired processing of temporal regularity in speech signals. Task performance correlated with standard disease severity and neurolinguistic measures. Across the patient cohort, performance on the temporal regularity task was associated with grey matter in the left supplementary motor area and right caudate, performance on the phoneme processing task was associated with grey matter in the left supramarginal gyrus, and performance on the prosodic predictability task was associated with grey matter in the right putamen.

**Conclusions:**

Our findings suggest that PPA syndromes may be underpinned by more generic deficits of auditory signal analysis, with a distributed cortico-subcortical neuraoanatomical substrate extending beyond the canonical language network. This has implications for syndrome classification and biomarker development.

**Electronic supplementary material:**

The online version of this article (doi:10.1186/s13195-017-0278-2) contains supplementary material, which is available to authorized users.

## Background

The primary progressive aphasias (PPAs) continue to present substantial problems of classification and diagnosis. A number of patients do not meet consensus diagnostic criteria for particular PPA syndromes [1], while the major syndromes show clinical and anatomical overlap [2]. Accumulating evidence suggests that abnormalities of speech processing in these ‘language-led’ dementias may reflect broader deficits of non-linguistic auditory signal decoding; indeed, presentations with progressive word deafness were among the first descriptions of PPA [3] and have since been expanded upon in some detail [4–13]. Abnormalities of non-verbal auditory processing have been most consistently documented in the canonical non-fluent variant (nfvPPA) and semantic variant (svPPA) syndromes of PPA. These syndromes have relatively distinct clinico-anatomical profiles [1, 14]: nfvPPA presents with impaired speech production and/or agrammatism associated with asymmetric, predominantly left-sided peri-Sylvian atrophy; while svPPA characteristically presents with vocabulary loss and impaired word comprehension associated with asymmetric anterior temporal lobe atrophy. Consistent with these syndromic profiles, nfvPPA is associated with more prominent deficits of early perceptual auditory analysis including impaired temporal (rhythm) perception, while svPPA is particularly associated with auditory associative deficits and impaired sound meaning [4–6, 9–13]. The processing of certain auditory information (such as higher-order spectrotemporal statistics) is affected in both syndromes [5, 13]: this may reflect a fundamental computational deficit affecting the linkage of perceptual and semantic auditory object data [15, 16]. However, most studies of auditory processing in PPA have focused on non-verbal sounds and elementary acoustic patterns, rather than the acoustic analysis of speech signals per se. Moreover, the brain substrates that mediate auditory processing in PPA largely remain to be defined.

Here we address the auditory decoding of speech signals and its neurological basis in patients with nfvPPA and svPPA relative to healthy older individuals. Rather than using non-verbal sounds, we sought to probe the interface of non-linguistic and linguistic processing by manipulating acoustic properties of stimuli based on sequences of spoken syllables. We manipulated three generic characteristics of these sequences: inter-syllabic temporal regularity, phonemic spectral structure and overall signal predictability (fundamental information content or entropy, as embodied in prosodic pitch contours). The targeted characteristics broadly sample the processing stages of early perceptual coding, auditory object representation and decoding of higher-order patterns; these are not linguistic features as such, but underpin the linguistic processing of spoken messages. Participants were required to make forced-choice psychoacoustic decisions on each of these stimulus properties, and neuroanatomical correlates of psychoacoustic performance were assessed using voxel-based morphometry (VBM) of patients’ brain MR images.

In a recent functional magnetic resonance imaging (MRI) study, we used stimuli based on these manipulations to delineate functional cerebral networks engaged in auditory speech signal decoding in patients with PPA syndromes [17]. In the present study, we set out to identify the critical structural neuroanatomical correlates of psychoacoustic performance on these speech signal characteristics. Drawing on previous neuropsychological evidence, we hypothesised that patients with nfvPPA (but not svPPA) would show impaired processing of temporal regularity [8, 12, 18], while both patient groups would show impaired processing of spectral structure and predictability (fundamental information content) of speech signals [5, 6, 10–12, 16, 19]. We further hypothesised based on previous neuroanatomical work that the processing of temporal regularity and signal predictability would correlate with grey matter in a distributed frontotemporal–subcortical network comprising the posterior temporal, medial prefrontal and striatal cortex [20–24], while the processing of phonemic spectral structure would correlate with grey matter in the temporo-parietal cortex [25–28].

## Methods

### Participants

Ten patients with nfvPPA (five females; mean age 71.2 ± 8.9 (SD) years) and nine patients with svPPA (three females; mean age 63.8 ± 4.6 years) were recruited consecutively via a specialist cognitive clinic. All patients fulfilled current consensus criteria for a probable or definite diagnosis of the relevant PPA syndrome [1] and this was corroborated by general neuropsychological assessment and brain MRI findings. No patient had radiological evidence of significant co-morbid cerebrovascular disease. Nineteen healthy older individuals (10 females; mean age 69.4 ± 4.5 years) with no history of neurological or psychiatric illness also participated. No participant had a history of clinically significant hearing loss; peripheral hearing function was assessed in all participants using pure tone audiometry (details shown in Additional file 1). Demographic, clinical and neuropsychological data for all participants are summarised in Table 1.

**Table 1:** Demographic, clinical and neuropsychological characteristics of participant groups

Characteristic	Controls	nfvPPA	svPPA
General demographic and clinical			
Number (male:female)	9:10	5:5	6:3
Age (years)	69.4 (4.5)	71.2 (8.9)	63.8 (4.6)*
Handedness (right:left)	18:1	8:2	8:1
Education (years)	15.8 (2.4)	14.8 (2.9)	14.9 (2.9)
MMSE (/30)	29.7 (0.6)	**25.6 (4.6)**	19.7 (9.1)*
Symptom duration (years)	–	4.8 (2.8)	5.3 (2.8)
PTA best ear (N:Mild:Mod)	10:7:0^b^	3:5:1^a^	5:4:0
Background neuropsychological functions			
General intellect: IQ			
WASI verbal IQ	125.9 (7.3)	**90.7 (21.4)** ^**a**^	**72.3 (18.9)**
WASI performance IQ	124.6 (2.5)	**101.2 (7.0)**	**102.1 (25.6)**
Episodic memory			
RMT words (/50)	49.3 (0.9)	**43.5 (6.3)**	**36.0 (8.0)** ^c,^*
RMT faces (/50)	45.2 (3.1)	**39.3 (6.1)**	**33.3 (6.8)** ^c^
Camden PAL (/24)	20.4 (3.3)	**15.8 (6.8)** ^**b**^	2.2 (3.7)*
Working memory			
WMS-R digit span forward (max)	7.2 (1.2)	**5.1 (0.8)** ^b^	6.0 (1.9)
WMS-III spatial span forward (max)	5.5 (0.8)^c^	**4.3 (1.1)** ^c,^^†^	5.4 (0.9)
Executive skills			
WASI Block Design (/71)	45.4 (11.9)	**24.2 (18.9)**	34.6 (24.2)
WASI Matrices (/32)	26.4 (4.1)	**17.4 (8.6)**	**19.3 (10.5)**
WMS-R digit span reverse (max)	5.6 (1.2)	**3.4 (0.9)** ^b^	4.4 (2.1)
WMS-III spatial span reverse (max)	5.4 (1.0)^c^	4.4 (1.5)^c^	4.9 (2.0)
Letter fluency (F: total)	16.8 (5.0)	**5.5 (5.8)** ^d^	**7.3 (6.5)**
Category fluency (animals: total)	23.6 (5.5)	**10.7 (4.3)** ^**d**^	**4.9 (5.8)**
Trails A (s)	34.5 (6.8)^a^	**86.9 (50.0)** ^**b**^	**48.8 (18.2)** ^**a**^
Trails B (s)	72.8 (22.1)^a^	**192.0 (96.9)** ^**d**^	123.9 (87.7)^a^
Other skills			
Graded difficulty arithmetic (/24)	15.3 (5.5)	**5.7 (3.6)** ^c^	11.2 (9.8)
VOSP Object Decision (/20)	19.2 (1.3)^a^	**15.1 (4.6)** ^**a**^	**16.8 (3.1)** ^**a**^
Neurolinguistic functions			
Auditory input processing			
PALPA-3 (/36)	35.8 (0.5)^c^	**34.0 (2.6)** ^**c**^	**32 (6.5)**
Word retrieval			
GNT (/30)	26.4 (2.5)	**17.0 (7.1)** ^**a**^	1.9 (4.6)*
Speech comprehension			
BPVS (/51)	49.5 (1.3)	**43.4 (5.7)**	10.1 (14.9)*
Concrete synonyms (/25)	24.1 (0.8)^c^	21.3 (4.7)^c^	14.6 (3.2)*
Abstract synonyms (/25)	24.3 (0.9)^c^	21.1 (5.1)^c^	**15.9 (3.5)** ^c,^*
PALPA-55 sentences (/24)	23.8 (0.6)^e^	22.1 (3.3)^c^	19.7 (6.8)
Speech repetition			
Polysyllabic words (/45)	44.4 (0.9)^e^	**33.2 (12.0)** ^**d**^	43.8 (1.6)
Experimental psychoacoustic tasks^f^			
Temporal regularity (/20)	19.5 (1.0)	**18.0 (2.3)**	18.6 (2.7)
Phonemic structure (/20)	18.8 (1.6)	**15.3 (3.4)**	**15.6 (1.6)**
Prosodic predictability (/20)	19.1 (1.8)	**14.0 (3.1)**	**15.0 (4.0)**

Values represent mean (standard deviation) scores. Raw scores are presented, with the maximum value possible indicated in parentheses, unless otherwise indicated. Significant differences (*p* < 0.05) from healthy control values are indicated in bold

*Significantly different (*p* < 0.05) from nfvPPA group

^**†**^Significantly different (*p* < 0.05) from svPPA group

^a^Reduced number of participants: *n* – 1

^b^Reduced number of participants: *n* – 2

^c^Reduced number of participants: *n* – 3

^d^Reduced number of participants: *n* – 4

^e^Reduced number of participants: *n* – 5

^f^See text for details

*BPVS* British Picture Vocabulary Scale, *Controls* healthy control group, *GNT* Graded Naming Test, *MMSE* Mini-Mental State Examination score, *Mild* mild hearing loss, *Mod* moderate hearing loss, *N* normal hearing, *nfvPPA* non-fluent variant primary progressive aphasia, *PAL* paired associates learning, *PALPA* Psycholinguistic Assessments of Language Processing in Aphasia, *PTA* pure tone audiometry, *RMT* Recognition Memory Test, *svPPA* semantic variant primary progressive aphasia, *VOSP* Visual Object and Space Perception Battery, *WASI* Wechsler Abbreviated Scale of Intelligence, *WMS* Wechsler Memory Scale

All participants gave informed consent. Ethical approval for the study was granted by the National Hospital for Neurology and Neurosurgery and the University College London Research Ethics Committees, in accordance with the Declaration of Helsinki.

### Experimental stimuli

For the experimental stimuli, we created sequences of spoken syllables consisting of consonant–vowel or vowel–consonant phoneme combinations. We chose the syllables ‘af’, ‘ba’, ‘da’, ‘mo’, ‘om’, ‘or’, ‘po’ and ‘ro’ for their high intelligibility and identifiability, based on pilot work in five young adult listeners (details shown in Additional file 1). Syllables were recorded in a standard southern English accent by a young adult male speaker. Using MATLAB R2012a (https://uk.mathworks.com/), syllables were concatenated with random ordering to form sequences each comprising 20 syllables of duration 240 ms and fundamental frequency 100 Hz, with intervening silent intervals. The overall sequence duration (7.65 seconds) and root mean square intensity were fixed across sequences. Different conditions were created by independently varying three sequence parameters: temporal regularity, phonemic structure and entropy (fundamental information content, a measure of signal unpredictability).

Temporal regularity was varied by altering the inter-syllabic interval such that this was either kept constant at 150 ms (isochronous condition) or randomly allocated in the range 50–250 ms around a mean of 150 ms (anisochronous condition) while maintaining the same overall sequence tempo. Phonemic structure was varied using a previously described procedure of spectral rotation [29]: this manipulation preserves overall acoustic spectro-temporal complexity and bandwidth but profoundly affects spectral detail, by inverting the acoustic frequency spectrum and thereby rendering the rotated signal unintelligible as human speech (listeners generally describe it as ‘alien’ or ‘computer speech’). We synthesised stimulus conditions in which the constituent syllables comprising each sequence were either all unrotated (natural) or all spectrally rotated (unintelligible). Speech signal predictability was varied as an index of fundamental information content or entropy of the syllable sequences: in classical information theory, signals with high fundamental information content (or entropy) have low predictability. We adapted a previously described procedure [21] to manipulate the overall predictability of the pitch contour of each syllable sequence. This procedure varied the fundamental frequency (pitch) of constituent syllables over a half-octave range, using a 20-note octave division that did not conform to Western musical intervals; pitch sequences (prosodic contours) were based on inverse Fourier transforms of f^*n*^ power spectra with values *n* = 0 (no correlation between consecutive syllable pitch values; the low signal predictability–high entropy condition) and *n* = 4 (high correlation between consecutive syllable pitch values, approaching a sine-wave contour; the high signal predictability–low entropy condition). It is important to note that this prosodic manipulation does not correspond to any single feature of natural prosody: rather, it taps into a generic statistical property of prosodic contours (the correlation structure of the syllabic pitch sequence) that is potentially relevant to many kinds of patterns in speech signals.

**Fig. 1:**
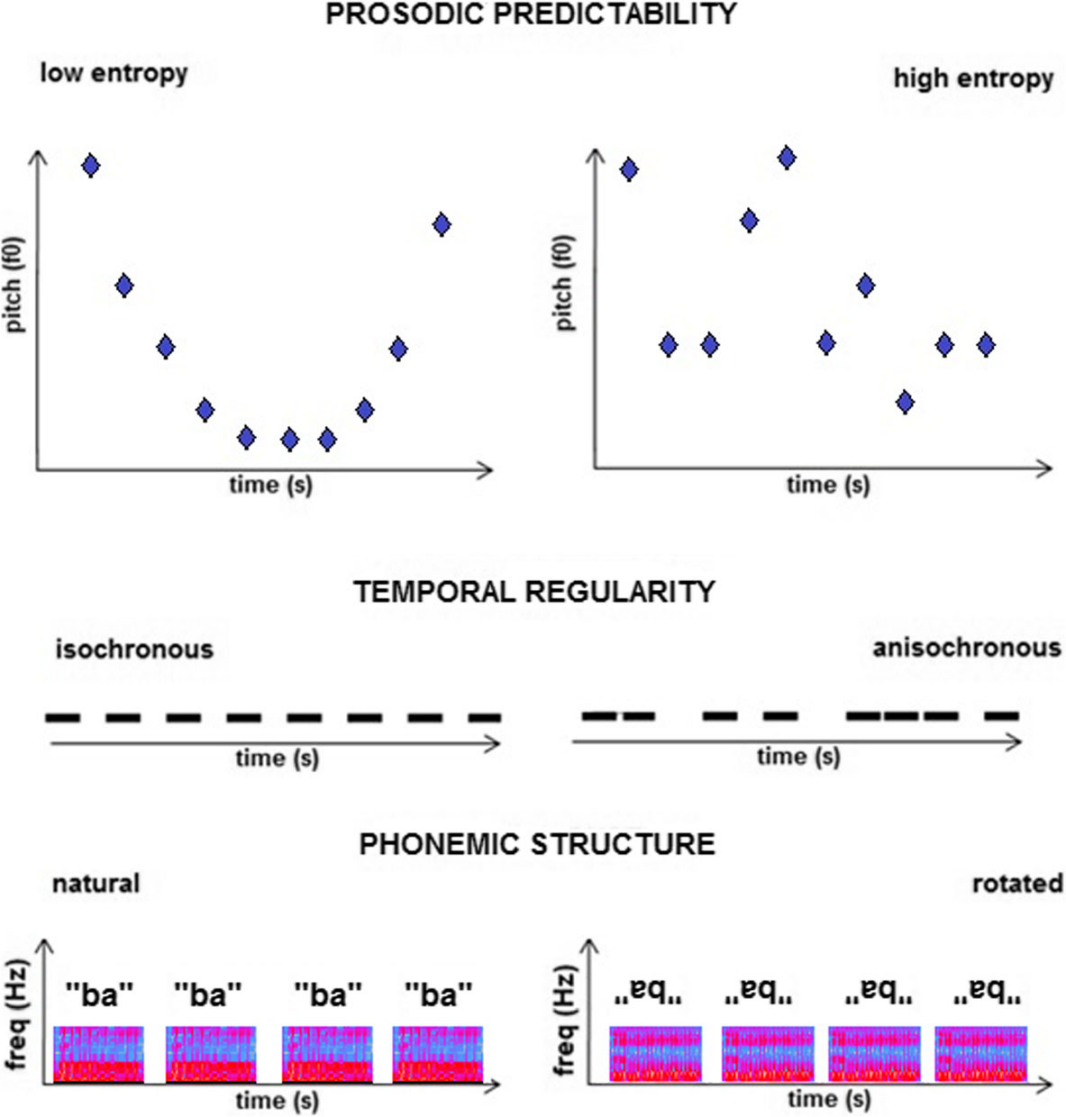
Schematic representations of stimulus manipulations used to create the conditions in the experiment (see text for details). *Top panels*: examples of high and low predictability (low and high entropy) sequences, based on degree of correlation between pitch (fundamental frequency, *f0*) of successive syllables (highly correlated and approaching a sine-wave prosodic contour in the low entropy condition; uncorrelated in the high predictability condition). *Middle panels*: examples of isochronous (temporally regular) and anisochronous (temporally irregular) sequences. *Bottom panels*: spectrograms for syllable sequences in the natural and spectrally rotated (unintelligible) conditions. *freq* frequency

The stimuli are schematised in Fig. 1; examples are provided in Additional files 2, 3, 4, 5, 6 and 7.

### Experimental psychoacoustic test procedure

These experimental stimuli formed the basis for three two-alternative, forced-choice psychoacoustic tasks, each probing a particular dimension of auditory processing. Separate tests were administered to assess pitch pattern analysis (predictable vs unpredictable sequences), temporal processing (regular vs irregular sequences) and phoneme detection (natural vs artificial (spectrally rotated, unintelligible) phonemes). Tests were administered in the same order to all participants: first, pitch pattern analysis; second, temporal processing; and third, phoneme detection. For each test, 20 stimuli (10 representing each of the two conditions of interest) were presented. For the test assessing processing of prosodic predictability, participants were asked to decide whether the sounds were arranged randomly or following a pattern; for the test assessing temporal processing, participants were asked on each trial to decide whether the sounds they heard came regularly or irregularly; and for the test assessing processing of phonemic structure, participants were asked to decide whether the sounds were made by a human or by a computer. Pictorial cue cards (see Additional file 8) were used as tools to ensure understanding of the task instructions in practice trials, before commencement of the test proper. On each trial, participants could respond verbally or by pointing to the relevant card.

Stimuli were presented in randomised order via a notebook computer running the Cogent v1.32 extension of MATLAB (www.vislab.ucl.ac.uk/cogent_2000.php). No feedback about task performance was given and no time limits were imposed. Participant responses were recorded for offline analysis.

### Analysis of clinical and behavioural data

Clinical and behavioural data were analysed using Stata® v14.1. Participant groups were compared on demographic and other clinical variables using two-tailed, two-sample *t* tests for continuous variables and chi-square tests for categorical variables. Non-parametric Mann–Whitney *U* tests were used to compare groups on neuropsychological parameters where residuals were non-normally distributed.

In order to compare groups for peripheral hearing function, we first created a composite pure tone average score based on the average volume (dB) required for tone detection at 500, 1000 and 2000 Hz, for each ear separately. Using data from the best ear for each participant, scores within the range of 0–25 dB were categorised as ‘normal’, scores of 26–40 dB were classified as ‘mild hearing loss’ and scores of 41–55 dB classified as ‘moderate hearing loss’. Based on these classifications, each participant’s hearing function was treated as a categorical variable and Fisher’s exact test was used to compare groups.

In separate regression (Spearman’s rank correlation) analyses over the participant cohort, we assessed experimental psychoacoustic task performance against background executive function (WASI Matrices score; a proxy for disease severity) and a standard measure of phoneme discrimination (PALPA-3 score).

For all tests, the statistical threshold *p* < 0.05 was accepted as the criterion of significance.

### Brain MRI acquisition and VBM

Volumetric brain MR images were acquired for all patients in a 3 Tesla Siemens Tim Trio MRI scanner, using a 32-channel receiver array head coil and a T1-weighted sagittal 3D magnetisation prepared rapid gradient echo (MPRAGE) sequence (TE = 2.9 ms, TI = 900 ms, TR = 2200 ms), with dimensions 256 mm × 256 mm × 208 mm and voxel size 1.1 mm × 1.1 mm × 1.1 mm.

For the VBM analysis, patients’ brain images were first pre-processed and normalised to MNI space using SPM12 software (http://www.fil.ion.ucl.ac.uk/spm/software/spm12/) and the DARTEL toolbox with default parameters running under MATLAB R2012a. Images were smoothed using a 6-mm full-width at half-maximum Gaussian (FWHM) kernel. To control for individual differences in total (pre-morbid) brain size, total intracranial volume was calculated for each participant by summing white matter, grey matter and cerebrospinal fluid volumes post segmentation. A study-specific average brain upon which to overlay statistical parametric maps was created by warping all patients’ native-space whole-brain images to the final DARTEL template and using the ImCalc function to generate an average of these images.

We firstly assessed disease-associated atrophy profiles in each patient group. Voxel intensity (grey matter volume) in each syndromic group separately was contrasted with the healthy control group, incorporating age and total intracranial volume as nuisance covariates. Statistical parametric maps were thresholded at peak-level *p* < 0.001 uncorrected for multiple voxel-wise comparisons over the whole brain, in order to delineate the extent of disease-associated atrophy in each patient group.

We assessed neuroanatomical correlates of experimental behavioural task performance in a separate analysis. Voxel intensity was modelled for the combined patient cohort as a function of performance on each of the experimental psychoacoustic tasks in a multiple regression design incorporating age, total intracranial volume, disease duration and group membership as nuisance covariates. An explicit brain mask was created using an automatic mask-creation strategy described previously [30]. Statistical parametric maps were thresholded at a peak-level *p* < 0.05 after family-wise error (FWE) correction for multiple voxel-wise comparisons within a pre-defined region of interest, based on neuroanatomical predictions from previous studies. Correlates of behavioural performance on the temporal regularity and prosodic predictability tests were assessed within a region comprising the bilateral posterior superior temporal gyrus, planum temporale, supramarginal gyrus, supplementary motor area, anterior cingulate and striatum [20–23]. Grey matter correlates of performance on the phoneme detection test were assessed with a more restricted sub-region comprising the left posterior superior temporal gyrus, planum temporale and supramarginal gyrus [25–28]. Anatomical regions were derived from Oxford–Harvard cortical maps [31] and are depicted in Additional file 9.

## Results

### General participant characteristics

Comparisons of general characteristics and neuropsychological performance between participant groups are summarised in Table 1.

Patient groups did not differ significantly from healthy controls in terms of gender, handedness or years in formal education (all *p* > 0.05). The svPPA group was significantly younger than both the healthy control (*p* = 0.005) and nfvPPA (*p* = 0.04) groups (accordingly, the effect of age as a nuisance covariate of group experimental psychoacoustic task performance was assessed separately). The two patient groups had comparable symptom duration (*p* = 0.7) and level of overall cognitive impairment (as indexed using Mini-Mental State Examination score; *p* = 0.09). Participant groups showed no significant differences in peripheral hearing (see Table 1).

### Experimental psychoacoustic task performance

**Fig. 2:**
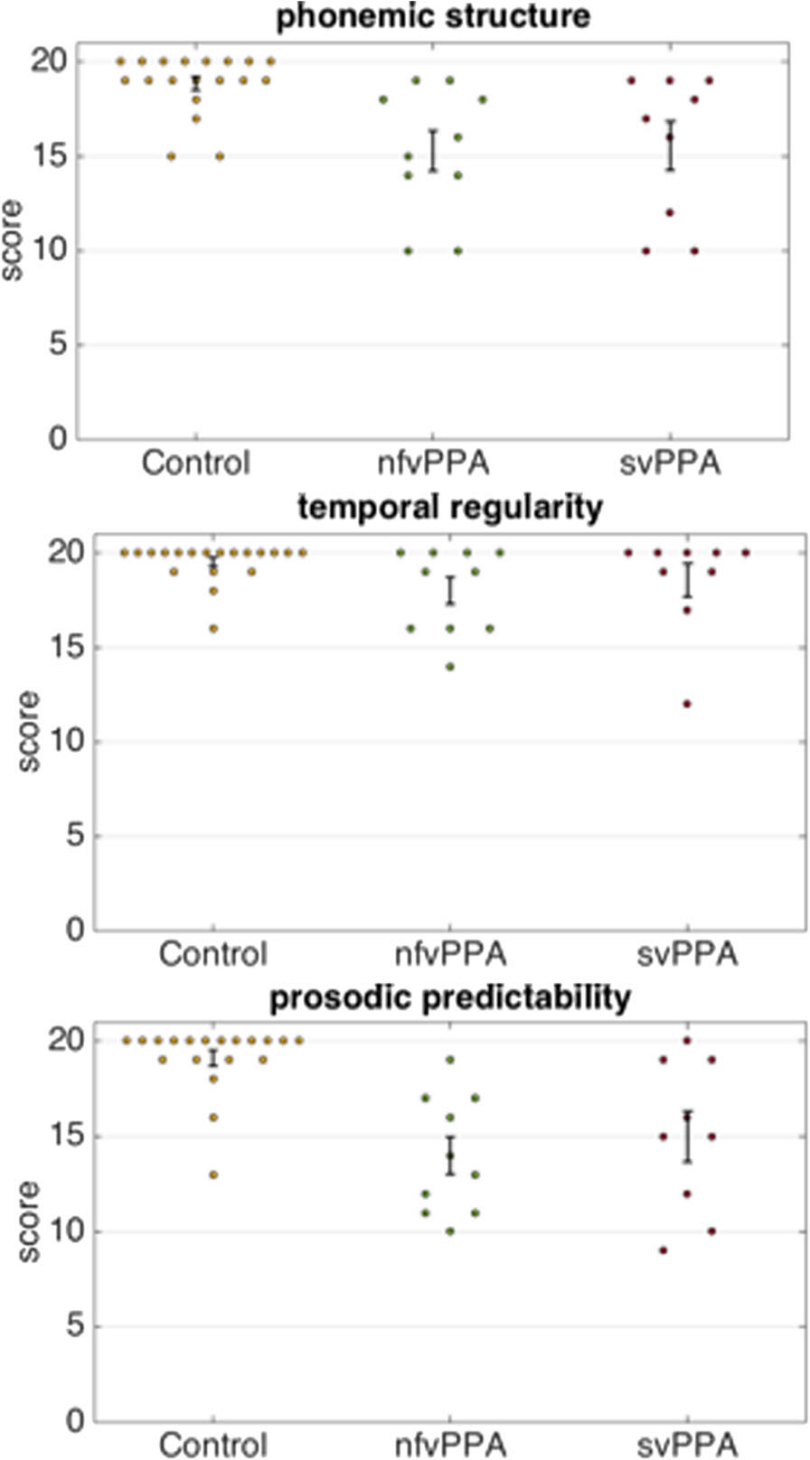
Plots of individual data for performance on each of the experimental psychoacoustic tasks, for each participant group. *Error bars* represent standard error of the mean. *Control* healthy control group, *nfvPPA* patient group with non-fluent primary progressive aphasia, *svPPA* patient group with semantic variant primary progressive aphasia

Group performance profiles on the experimental psychoacoustic tasks are summarised in Table 1 and individual data are plotted in Fig. 2. On the tests of phoneme detection and prosodic predictability analysis, both patient groups performed significantly worse than the healthy control group (all *p* < 0.05). On the test of temporal regularity processing, the nfvPPA group performed significantly worse than the healthy control group (*p* = 0.03) whereas the performance of the svPPA group did not differ significantly from controls (*p* = 0.07). This pattern of results was not altered by incorporating age as a nuisance covariate.

Performance on each of the experimental psychoacoustic tasks correlated significantly with a standard measure of background executive capacity (WASI Matrices score; all *p* < 0.001), an index of overall disease severity. Performance on the experimental phoneme detection task correlated significantly with a standard measure of phoneme discrimination ability (PALPA-3 score; *p* = 0.001). The correlation between PALPA-3 score and prosodic predictability score was also significant (*p* = 0.004), while the correlation between PALPA-3 score and temporal regularity score was not significant (*p* = 0.06).

### Neuroanatomical data

**Fig. 3:**
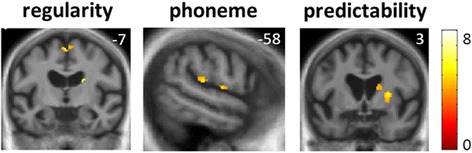
Statistical parametric maps of regional grey matter volume positively associated with performance on speech signal analysis tasks (assessing processing of temporal regularity, phonemic spectral structure and prosodic predictability, respectively) in the combined patient cohort. Maps are rendered on sections of the group mean T1-weighted MR image in MNI space, thresholded at *p* < 0.001 uncorrected for multiple voxel-wise comparisons over the whole brain for display purposes (areas shown were significant at *p* < 0.05_FWE_ for multiple comparisons within a pre-specified neuroanatomical region of interest; see Additional file 9). Colour bar (*right*) codes voxel-wise *t* values. The plane of each section is indicated using the corresponding MNI coordinate (mm); the right cerebral hemisphere is shown on the right in the coronal sections (Colour figure online)

Statistical parametric maps of grey matter regions associated with performance on the experimental psychoacoustic tasks in the combined patient cohort are shown in Fig. 3 and maps of disease-associated atrophy are shown in Additional file 10; local maxima of grey matter change correlated with experimental psychoacoustic task performance are summarised in Table 2 and local maxima for disease-related atrophy are summarised in Additional file 11.

**Table 2:** Structural neuroanatomical associations of speech signal analysis in the patient cohort

Contrast	Region	Side	Cluster (voxels)	Peak (mm)			*t* value	*p* value
				*x*	*y*	*z*		
Temporal regularity	Supplementary motor^a^	Left	427	–2	–9	63	7.93	0.016
	Caudate	Right	216	16	–2	20	7.02	0.042
Phonemic structure	Supramarginal gyrus^a^	Left	12	–58	–28	14	5.53	0.026
Prosodic predictability	Putamen	Right	289	28	0	6	7.01	0.035

Summary of statistically significant positive associations between grey matter volume and performance on psychoacoustic tasks to assess the temporal regularity, phonemic structure and prosodic predictability of experimental speech stimuli (see text for details), based on a voxel-based morphometric analysis of brain magnetic resonance images for the combined patient cohort. All values were significant at *p* < 0.05_FWE_ within a pre-specified neuroanatomical region of interest (see Additional file 9); coordinates of local maxima are in MNI standard space

*FWE* family-wise error

^a^Local maximum coincident with regional disease-related grey matter atrophy in the non-fluent variant primary progressive aphasia group (see Additional file 11)

Compared with the healthy control group, each syndromic group exhibited the anticipated profile of disease-associated grey matter loss (Additional file 10). The nfvPPA group had bilateral, predominantly fronto-insular atrophy that was more marked in the left cerebral hemisphere. The svPPA group showed asymmetric atrophy predominantly involving the antero-mesial and inferior temporal lobes, again more marked in the left cerebral hemisphere.

Performance on the task assessing temporal regularity in speech signals was positively associated with grey matter volume in the left supplementary motor area and right caudate (both *p* < 0.05_FWE_ within the pre-specified region of interest). Performance on the task assessing phoneme detection was associated with grey matter volume in the left supramarginal gyrus (*p* < 0.05_FWE_ within the pre-specified region of interest). Performance on the task assessing prosodic predictability was associated with grey matter volume in the right putamen (*p* < 0.05_FWE_ within the pre-specified region of interest).

## Discussion

We have demonstrated behavioural and neuroanatomical correlates of the defective analysis of generic speech signal attributes in two canonical PPA syndromes. In line with previous neuropsychological evidence concerning the processing of non-verbal sounds in PPA [5, 6, 9–13], processing of speech signal temporal regularity (an early perceptual property) was impaired in the patient group with nfvPPA, while processing of phonemic spectral structure and prosodic predictability (higher-order auditory properties) was impaired in both patient groups. Taken together, our findings substantiate an emerging picture of more generic, extra-linguistic deficits that may contribute to the hallmark neurolinguistic syndromes of PPA. The psychoacoustic deficits identified in our patient cohort had separable structural neuroanatomical substrates within distributed cerebral cortico-subcortical networks previously implicated in the analysis of auditory object and multimodal sensory information [17, 20–28].

Impaired processing of auditory rhythm and a neuroanatomical correlate in the supplementary motor cortex have been reported previously in nfvPPA [12, 24]: our findings show that this mechanism extends to speech signals and support a link between impaired perception and production of speech in these patients. In addition to any deficit of motor speech planning, impaired tuning, monitoring and rehearsal of own speech output might contribute to impaired production of lexical stress and prosody in patients with nfvPPA [12, 24, 32]. Supplementary motor cortex mediates the tracking and integration of prosodic and syntactical rhythms in the healthy brain [33]; it has been proposed that speech apraxia in nfvPPA may at least in part reflect dysfunctional integration of temporal perceptual and speech output processes [12, 24, 34]. An additional correlate of temporal regularity processing was identified here in the caudate nucleus, consistent with previous work implicating the striatum in tracking of speech and other stimuli with extended temporal structures [35]. Our findings corroborate previous formulations of nfvPPA as an essentially fronto-striatal disorder [36, 37].

The phonemic spectral processing deficit exhibited by both patient groups reflects impaired representation of auditory object features: whereas phonemes constitute a specialised category of auditory objects, an analogous deficit has been demonstrated previously to affect a range of non-verbal sounds in both nfvPPA and svPPA [5]. While linguistic phonological impairment is well recognised as a feature of nfvPPA, the present findings in the context of previous work suggest that phonemic deficits may be underpinned by a generic defect of auditory apperceptive function [5–8, 12, 18]. The neuroanatomical correlate of impaired phoneme detection in our patient cohort was localised to the left supramarginal gyrus: this temporo-parietal junctional zone has been identified previously as a phonological processing hub in the healthy brain [38] and a seat of apperceptive discrimination of non-linguistic sound objects such as human voices [7]. Moreover, PPA syndromes show convergent involvement of this region [2]. Although linguistic phonological impairment is not a defining feature of svPPA, this syndromic group has been shown to have deficits extending to the perceptual analysis of sounds [5, 12]: this might be parsimoniously interpreted as evidence for impaired top-down integration of auditory object properties into conceptual representations, in keeping with current computational models of semantic cognition [16].

Both syndromic groups here showed impaired analysis of prosodic predictability, an index of the fundamental, non-linguistic information content of speech signals. This deficit had a neuroanatomical correlate in the right putamen, corroborating work in the healthy brain implicating the striatum in tracking and probabilistic coding of sensory signals [21, 35, 39–41]. This finding is in line with previous evidence for impaired extraction of global statistical regularities in auditory signals in both nfvPPA and svPPA [5]: a core deficit of this kind might potentially disrupt the decoding of syntactic, prosodic and musical patterns in nfvPPA [9, 11] and computation of coherent auditory object concepts in svPPA [15, 16].

## Conclusions

From a clinical perspective, our findings show that generic auditory processing deficits in PPA syndromes extend to the processing of speech signals and suggest that such deficits may correlate with overall disease severity as well as standard measures (here, phonemic discrimination) of linguistic competence in these syndromes. With respect to the nosology of PPA, these findings suggest that certain measures of speech signal analysis (such as temporal coding) may stratify syndromes, whereas other measures (such as spectral and statistical coding) may cross conventional syndrome boundaries. These behavioural measures capture regional atrophy within a distributed fronto-temporo-parietal network that overlaps but extends beyond canonical language areas (compare Table 2 and Additional file 11), involving striatal structures implicated in non-verbal pattern decoding. This study requires substantiation in larger patient cohorts, ideally with longitudinal tracking of deficits and, ultimately, histopathological correlation. The relations between linguistic and pre-linguistic impairment in PPA will only be fully defined through more comprehensive neuropsychological correlation and functional neuroimaging techniques that address underlying neural mechanisms directly [17]. We regard the present work as a prima-facie case for the systematic exploration of non-verbal signal processing functions in PPA, with a view to re-evaluating conventional syndrome definitions and new biomarker discovery.

## Additional files

Additional file 1: Presents additional methodological information: procedure for testing peripheral hearing and selection of experimental stimuli. (PDF 157 kb)

Additional file 2: Is a sound file presenting a regular sequence stimulus example. (WAV 658 kb)

Additional file 3: Is a sound file presenting an irregular sequence stimulus example. (WAV 651 kb)

Additional file 4: Is a sound file presenting a high predictability sequence stimulus example. (WAV 670 kb)

Additional file 5: Is a sound file presenting a low predictability sequence stimulus example. (WAV 670 kb)

Additional file 6: Is a sound file presenting a natural phoneme sequence stimulus example. (WAV 628 kb)

Additional file 7: Is a sound file presenting a rotated phoneme sequence stimulus example. (WAV 632 kb)

Additional file 8: Is a figure showing visual aids. (PDF 119 kb)

Additional file 9: Is a figure showing small volume corrections. (PDF 125 kb)

Additional file 10: Is a figure showing statistical parametric maps of disease-associated grey matter atrophy in each patient group relative to healthy controls, based on a voxel-based morphometric analysis. (PDF 49 kb)

Additional file 11: Is a table presenting neuroanatomical associations of disease-related grey matter atrophy. (PDF 140 kb)

## Abbreviations

MRI: Magnetic resonance imaging
nfvPPA: Non-fluent variant primary progressive aphasia
PALPA: Psycholinguistic Assessments of Language Processing in Aphasia
PPA: Primary progressive aphasia
svPPA: Semantic variant primary progressive aphasia
VBM: Voxel-based morphometry
WASI: Wechsler Abbreviated Scale of Intelligence
