# Supplementary material for: Behavioural and neuroanatomical correlates of auditory speech analysis in primary progressive aphasias
Source: Alzheimers Res Ther. 2017 Jul 27;9:53. doi: 10.1186/s13195-017-0278-2 (PMC5531024; doi:10.1186/s13195-017-0278-2)
Supplement: Supplementary file 1 — Presents additional methodological information: procedure for testing peripheral hearing and selection of experimental stimuli. (PDF 157 kb) [file 13195_2017_278_MOESM1_ESM.pdf]

Additional file 1. Additional methodological information.

Procedure for assessing peripheral hearing

Pure tone audiometry was performed using an Otovation Roto audiometer (www.otovation.com) in a quiet room. Five frequency levels were tested (500, 1000, 2000, 4000, 6000 Hz). At each frequency, the participant was played three tones, starting at 20dB. If the participant indicated correctly that they had heard at least two of the three tones, this was recorded as the threshold for that frequency; if not, the level was increased in increments of 5dB up to 70dB. Hearing was assessed in both ears for each participant.

Selection of experimental stimuli

In order to select a set of carrier syllables for the experimental manipulations, in pilot work we presented 32 recorded VC and CV syllables to five young healthy listeners (3 female, 2 male, aged 23 – 32 years). Each pilot participant was asked to transcribe each of the syllables. The syllables that were most accurately transcribed across the pilot group were selected for incorporation in the experimental stimuli.
