# Supplementary material for: Behavioural and neuroanatomical correlates of auditory speech analysis in primary progressive aphasias
Source: Alzheimers Res Ther. 2017 Jul 27;9:53. doi: 10.1186/s13195-017-0278-2 (PMC5531024; doi:10.1186/s13195-017-0278-2)
Supplement: Supplementary file 8 — Is a figure showing visual aids. (PDF 119 kb) [file 13195_2017_278_MOESM8_ESM.pdf]

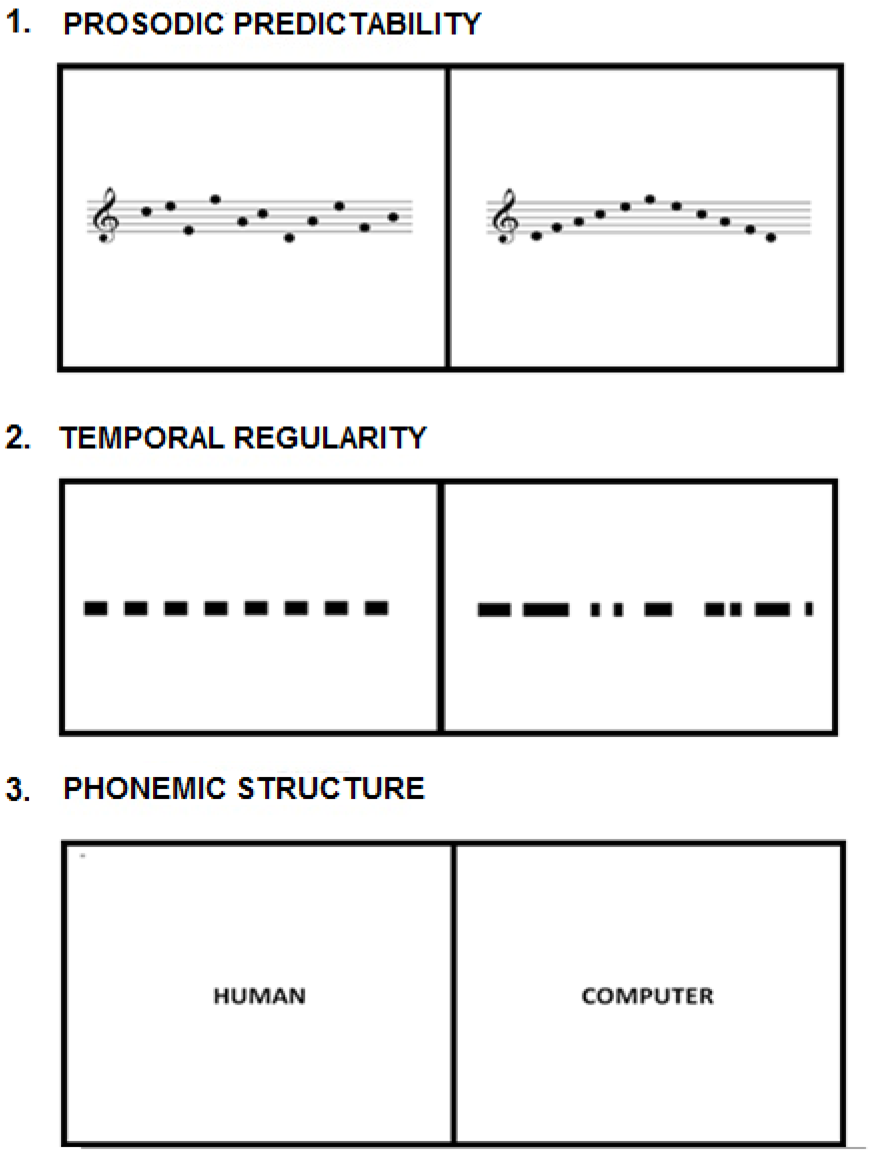

Additional file 8. Pictorial cue cards were used as tools to ensure understanding of each of the experimental psychoacoustic tasks. 1) For the test assessing processing of prosodic predictability (top panels), participants were asked to decide whether the sounds were arranged randomly or following a pattern. 2) For the test assessing temporal processing (middle panels), on each trial participants were asked to decide whether the sounds they heard came regularly or irregularly. 3) For the test assessing processing of phonemic structure (bottom panels), participants were asked to decide whether the sounds were made by a human or by a computer. On each trial, participants could respond verbally or by pointing to the relevant card.
