# Supplementary material for: Behavioural and neuroanatomical correlates of auditory speech analysis in primary progressive aphasias
Source: Alzheimers Res Ther. 2017 Jul 27;9:53. doi: 10.1186/s13195-017-0278-2 (PMC5531024; doi:10.1186/s13195-017-0278-2)
Supplement: Supplementary file 9 — Is a figure showing small volume corrections. (PDF 125 kb) [file 13195_2017_278_MOESM9_ESM.pdf]

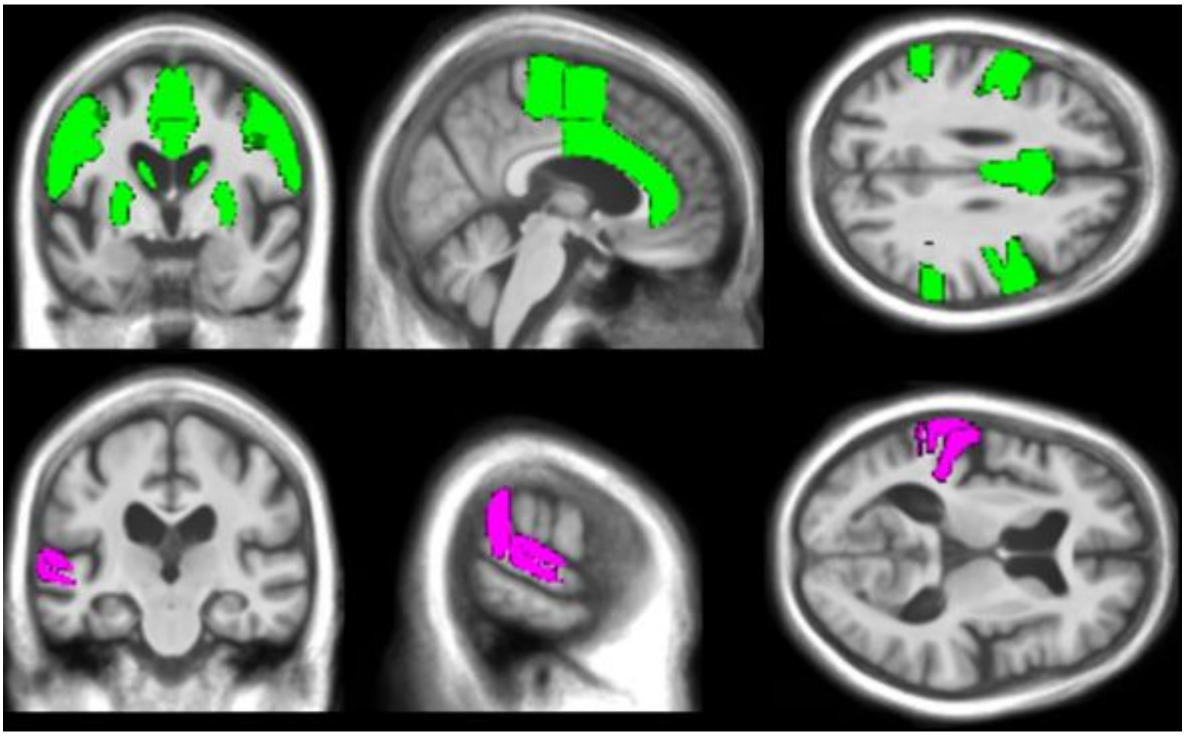

Additional file 9. Representative sections of neuroanatomical volumes used for multiple voxel-wise comparisons corrections in region-of-interest analyses based on prior anatomical hypotheses (see text). Bi-hemispheric regions of interest are rendered on sections of the mean normalised brain template for the patient cohort. For the contrasts assessing temporal processing and prosodic predictability processing, the anatomical region of interest (green, above) comprised bilateral posterior superior temporal gyrus/sulcus, planum temporale, supramarginal gyrus. striatum (caudate and putamen), supplementary motor cortex and anterior cingulate. For the contrast assessing phonemic processing, the anatomical region for small volume correction (lilac, below) was a subregion comprising left posterior superior temporal gyrus/sulcus, planum temporale and supramarginal gyrus.
