# Supplementary material for: Behavioural and neuroanatomical correlates of auditory speech analysis in primary progressive aphasias
Source: Alzheimers Res Ther. 2017 Jul 27;9:53. doi: 10.1186/s13195-017-0278-2 (PMC5531024; doi:10.1186/s13195-017-0278-2)
Supplement: Supplementary file 10 — Is a figure showing statistical parametric maps of disease-associated grey matter atrophy in each patient group relative to healthy controls, based on a voxel-based morphometric analysis. (PDF 49 kb) [file 13195_2017_278_MOESM10_ESM.pdf]

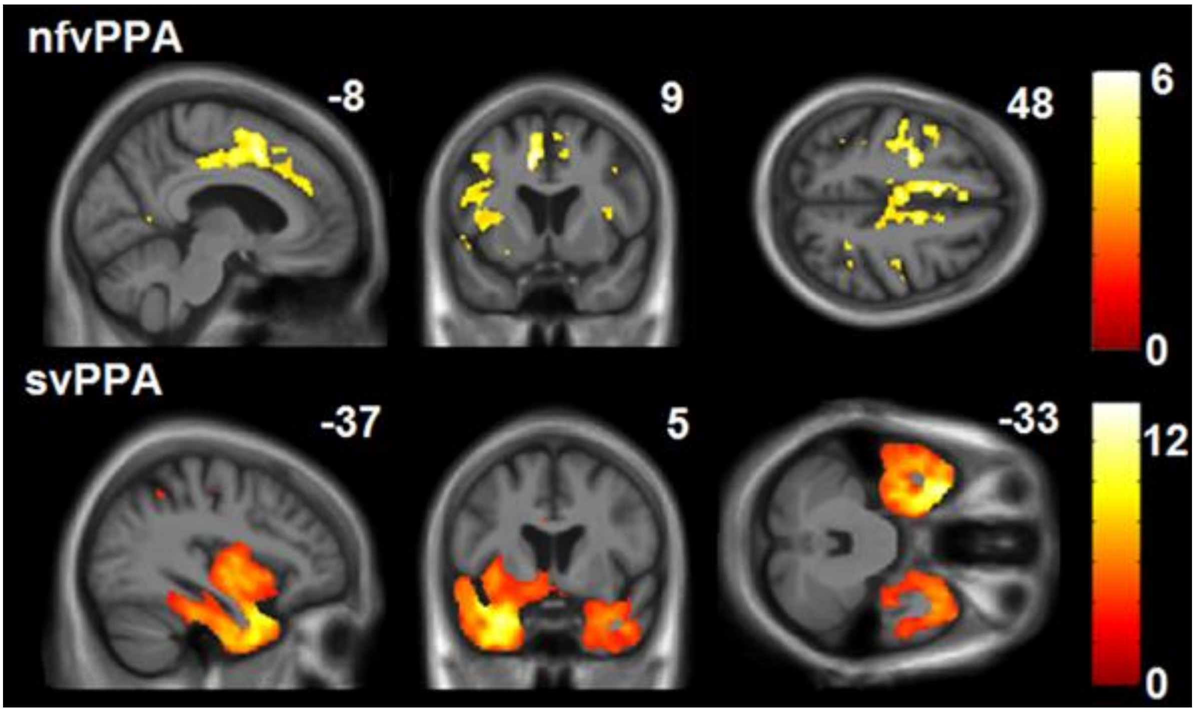

Additional file 10. Statistical parametric maps of disease-associated grey matter atrophy in each patient group relative to healthy controls, based on a voxel-based morphometric analysis. Maps are rendered on representative sections of the group mean T1-weighted MR image in MNI space, thresholded at $p < 0.001$ uncorrected for multiple voxel-wise comparisons over the whole brain. The colour bars (right) code voxel-wise t-values of grey matter differences. The plane of each section is indicated using the corresponding MNI coordinate (mm); the left cerebral hemisphere is shown on the left in the coronal sections and on top in the axial sections. nfvPPA, patient group with nonfluent variant primary progressive aphasia; svPPA, patient group with semantic variant primary progressive aphasia.
