# Supplementary material for: Behavioural and neuroanatomical correlates of auditory speech analysis in primary progressive aphasias
Source: Alzheimers Res Ther. 2017 Jul 27;9:53. doi: 10.1186/s13195-017-0278-2 (PMC5531024; doi:10.1186/s13195-017-0278-2)
Supplement: Supplementary file 11 — Is a table presenting neuroanatomical associations of disease-related grey matter atrophy. (PDF 140 kb) [file 13195_2017_278_MOESM11_ESM.pdf]

Additional file 11. Neuroanatomical associations of disease-related grey matter atrophy

**Region**	**Side**	**Cluster** (voxels)	**Peak** (mm)			**t-value**
			**x**	**y**	**z**	
**Nonfluent primary progressive aphasia**						
Supplementary motor cortex*	L	2581	-8	9	48	5.97
Insula/ precentral gyrus	L	4903	-38	14	2	5.80
Lingual gyrus	R	104	21	-48	-4	5.19
Lingual gyrus	L	595	-15	-56	-2	5.05
Middle frontal gyrus	R	108	45	12	34	5.00
Fusiform gyrus	L	185	-28	-12	-33	4.90
Inferior temporal gyrus	L	376	-56	-63	-15	4.87
Supplementary motor cortex	R	141	12	3	60	4.84
Fusiform gyrus	R	279	28	-30	-24	4.78
Thalamus	L	101	-20	-32	-3	4.65
Precentral gyrus	R	304	40	-15	52	4.49
Supramarginal gyrus	R	193	57	-44	39	4.18
Supramarginal gyrus*	L	136	-40	-42	38	4.01
**Semantic variant primary progressive aphasia**						
Temporal pole	L	29614	-33	14	-32	13.82
Temporal pole	R	8845	39	9	-34	7.79
Anterior cingulate	L	103	-4	-3	33	4.71
Anterior cingulate (separate locus)	L	406	-4	24	32	4.04

The Table summarises the distribution of significant disease-related regional grey matter loss, comparing each syndromic group with the healthy control group in a voxel based morphometric analysis. All values shown were significant at a lenient threshold $p<0.001$ uncorrected over the whole brain volume; clusters >100 voxels in size are included and coordinates of local maxima are in MNI standard space; *region also identified as a significant association of experimental psychoacoustic task performance (see Table 2).
